# Floating sphere assay: A rapid qualitative method for microvolume analysis of gelation

**DOI:** 10.1371/journal.pone.0266309

**Published:** 2022-07-08

**Authors:** Uma Jingxin Tay, Megan Goh, Jeralyn Ching Wen Hui, Prakash Arumugam

**Affiliations:** 1 Singapore Institute of Food and Biotechnology Innovation, Singapore, Singapore; 2 Nanyang Technological University, School of Biological Sciences, Singapore, Singapore; University of South Carolina, UNITED STATES

## Abstract

A huge, unprecedented demand for gelatin coupled with its implications on global sustainability has resulted in the need to discover novel proteins with gelling attributes for applications in the food industry. Currently used gelation assays require large sample volumes and thus the screening for novel gelling proteins is a formidable technical challenge. In this paper, we report the ‘Floating Sphere Assay’ which is a simple, economical, and miniaturized assay to detect minimum gelling concentration with volumes as low as 50 μl. Results from the Floating Sphere Assay are consistent with currently used methods for gelation tests and accurately estimate the Minimum Gelling Concentrations (MGCs) of gelatin, κ-carrageenan and gellan gum. The assay was also able to differentiate the strengths of strong and weak gellan gum gels prepared at pH 3.5 and pH 7.0 respectively. The Floating Sphere Assay can be utilized in high-throughput screens for gelling proteins and can accelerate the discovery of gelatin substitutes.

## Introduction

Gelling agents are used in a wide variety of convenient foods including pastas, salad dressings, yoghurt, ice-creams, jams, low calorie meals as well as restructured foods. A large proportion of gelling agents are polysaccharides like pectin, Sodium alginate, carrageenan and gellan gum. Gelatin is the only member of the protein family that is used in food gels. Polysaccharides are soluble dietary fibers and thus would be fermented by gut bacteria. Excessive fermentation leads to undesirable side effects such as flatulence, bloating, stomach discomfort, diarrhea and constipation [1]. Consequently, using protein-based gelling agents such as gelatin might be a preferable alternative for individuals prone to irritable bowel syndrome and colitis. Apart from being a widely used food ingredient, gelatin is increasingly adopted to constitute cell culture scaffold in engineering cell-cultured meat by virtue of its biocompatibility, biodegradability and low immunogenicity [2]. Consequently, the high reliance on gelatin which already has a market volume of 620.6 kilotons in 2019, is expected to continue growing at a volume-based Compound Annual Growth Rate of 5.9% between 2020 to 2027 [3].

Present reliance on gelatin is however unsustainable. Gelatin is mainly sourced from bones, skins, and tendons of bovines and pigs and to a lower extent from fish and poultry [3]. However, animal farming is inherently inefficient, straining the environment, public health and food security. Moreover, there is a rapid increase in veganism and vegetarianism across the world. Thus, the need for identifying plant-based protein alternatives for gelatin cannot be exaggerated.

It is possible to envisage two kinds of screening strategies for gelling proteins. In the first ‘activity-guided approach’, protein extracts from food-grade systems are fractionated and the gelling ability of individual protein fraction is assessed. In the second ‘candidate protein approach’, specific proteins from food-grade systems are expressed in hosts such as *E*. *coli* and purified, and the gelling ability of purified protein solutions is tested. Gelatin forms gels in the concentration range of about 20–100 mg/ml [4]. Assuming a similar concentration range for gelatin substitutes, large amounts of proteins would be required for performing such screens. Moreover, it will be necessary to sample a range of buffer and pH conditions for gelation screens. In the ‘activity-guided approach’, the gelling abilities of only the highly expressed proteins would be feasible to test and even this will require concentrating the protein fractions to small volumes. In the ‘candidate protein approach’, efficient expression and purification of the protein is essential. It is therefore highly desirable to develop miniaturized assays that require minimal solution volumes for assessing gelation.

**Fig 1:**
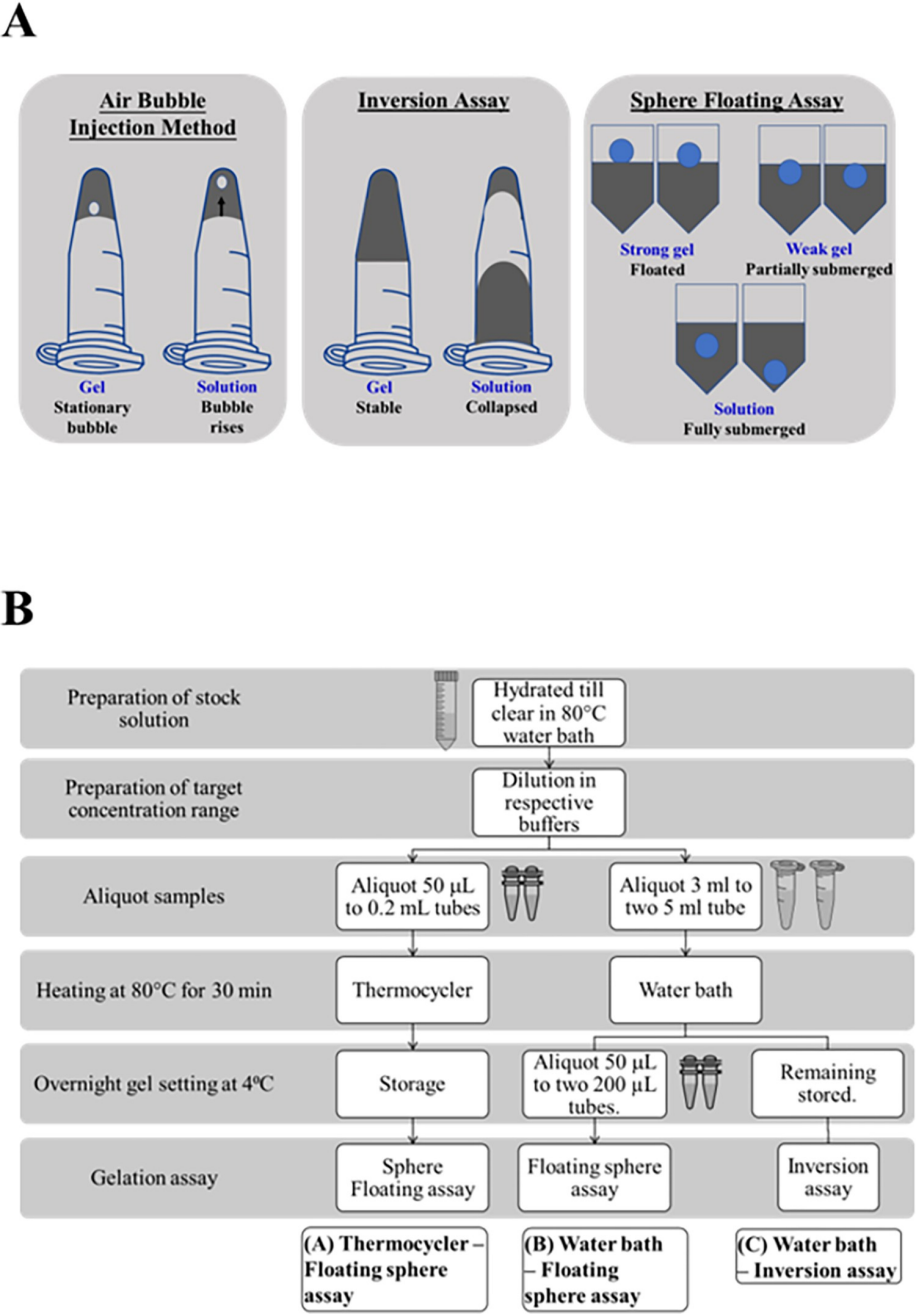
Floating sphere assay for assessing gelation. (A) Comparison of the proposed Floating Sphere Assay against conventional approaches. (B) Outline of the experimental strategy for Floating Sphere Assay.

The most widely used qualitative assay for gelation is the so-called ‘Inversion Assay’. In this assay, the tube containing a given sample is inverted to test if the solution flows or not (Fig 1A). If the solution does not flow, it is considered to have formed a gel. If the solution flows, it would indicate absence of gelation. Minimum Gelling Concentration (MGC) corresponds to the lowest concentration of the gelling agent at which the solution stays firm when tubes are inverted [5, 6]. However, this assay is not suitable for gelation screens with small solution volumes. For example, 500 μL solutions (non-gels) were observed to resist flow in 1.5 ml tubes [7]. As solutions resist flow presumably due to surface tension and adhesive forces between tube and the molecules, gelation tubes require a wide mouth, necessitating large sample volumes for the assay.

As an alternative to the Inversion Assay, Hughes and co-workers used an air bubble injection method–a bubble is introduced to samples (~500 μl) in inverted 1.5 ml microfuge tubes and gelation is detected by tracking the bubble [7]. If the bubble rises to the top of the tube, it is a solution and if the bubble remains at the liquid surface, it is a hydrogel (Fig 1A). However, injecting a bubble without fracturing the gel will be a challenge for brittle hydrogels. Fragmentation of the gel would be particularly pronounced with small sample volumes. Moreover, tracking the movement of the air bubble might not be straightforward.

‘Falling sphere viscometry’ is another assay which measures viscosity of solutions based on terminal velocity measurements of a ball dropped into the solution via Stokes equation [8, 9]. This technique has also been used to study gelation behavior of solutions [10–12]. However, these studies involved use of large sample volumes, and tracking sphere’s movement through the solution, which limits its application in high-throughput screens. Moreover, a parallel comparison of these afore-mentioned methods has not been done thus far.

In this paper, we report a simple miniaturized gelation assay referred to as the ‘Floating Sphere Assay’ that is adapted from the Falling Sphere Viscometry. This assay requires sample volumes as low as 50 μl and can be adapted for high throughput approaches (Fig 1A). On dropping the ball onto the solution, we assessed whether it floats on top (i.e. less than 25% of the bead has penetrated into the sample); partially submerges (more than 25% of the bead has penetrated) or fully submerges (the bead has sunk below the surface of the sample).

The Floating Sphere Assay can accurately determine the MGC of widely used gelling agents namely gelatin, potassium-supplemented κ-carrageenan and calcium-supplemented low acyl gellan. Gelatin is dominated by repeating motifs or glycine-(hydroxyproline or proline)-proline. On cooling, gelatin partially returns from disordered coils to polyproline II helix, resembling that of collagen. Around three regions of the helix generally interact. These regions transit from intra to inter-helix at higher concentrations, forming junction zones that induce gelation [13]. In contrast, gellan and κ carrageenan are linear anionic polysaccharides. Gellan comprises of tetrasaccharide repeats of [→3)-*β*-D- glucose-(1→4)-*β*-D-glucuronic acid -(1→4)-*β*-D-glucose-(1 → 4)-*β*-L-rhamnose-(1→)]. On cooling, gellan transits from disordered coils to double helices. Removal of acetyl substituents in the low acyl variant greatly reduces entropic and steric hindrance of the low acyl gellan gum helices to aggregation which is required for gelation. Aggregation of the double helices can be promoted by reducing electrostatic repulsion between glucuronate carboxylate groups through introducing cations (e.g. calcium) or lowering the pH. When calcium is incorporated, coordination binding between calcium and gellan gum further facilitates aggregation of the double helices [14]. κ-carrageenan comprises of disaccharide repeats of [→3)-*β*-D-galactose 4-sulfate-(1→4)-3,6-anhydro-α-D-galactose-(1→)]. Like gellan gum, κ-carrageenan undergoes coil-helix transition in the presence of potassium which coordinate the sulphate groups of κ-carrageenan, and aggregate into gel networks [15].

Overall, the results of gelatin, potassium κ-carrageenan and calcium-low acyl gellan from Floating Sphere Assay show good concordance with results from the widely used Inversion Assay. Moreover, the Floating Sphere Assay was able to distinguish the strength of strong and weak gellan gum gels prepared at pH 3.5 and 7.0 respectively.

## Material and methods

### Preparation of gels

Stock solutions of the gelling agents were prepared by hydrating the respective powders in their corresponding gelation buffers prepared using Milli-Q Water. Calcium κ-carrageenan was prepared by hydrating κ-carrageenan (22048, Sigma-Aldrich, Germany) in 200 mM KCl solution to form a 2% solution [15, 16]. Calcium-gellan gum was prepared by hydrating low acyl gellan gum (Kelcogel, CP Kelco, USA) in 5 mM CaCl_2_ to form a 2% solution [14]. Whereas gellan gum of pH 3.5 and 7.0 were prepared by hydrating low acyl gellan gum in respective buffers of pH 3.5 ± 0.1 and 7.0 ± 0.1 to form a 2% solution. Buffers were made by adjusting Milli-Q water using 5 M NaOH and 80% L-(+)-Lactic acid (27715, Sigma-Aldrich, Germany). Gelatin was prepared by hydrating porcine gelatin (G1890, Sigma-Aldrich, Germany) to form a 5% solution. Stock solutions were prepared by heating in an 80°C water bath under shaking at 90 rpm (SW22, JULABO GmbH, Germany) [14, 15]. The corresponding gelation buffer was similarly heated in the 80°C water bath and used for diluting the stock solution. For gelatin, a concentration range of 0.625%– 5.0% (0.625%, 1.25%, 2.50% and 5.0%) was used. For all other gelling agents, a concentration range of 0.0625–2.0% (0.0625%, 0.125%, 0.25%, 0.50%, 1.0%, 1.5% and 2.0%) was used.

For each specified concentration of the gelling agent, 3 ml was aliquoted to each of the duplicate 5.0 mL tubes (Eppendorf Tubes^®^, Germany) while 50 μL was aliquoted to each of the duplicate 200 μL Polymerase Chain Reaction (PCR) tubes (Axygen^®^, Corning Incorporated, USA). Samples in the 5 mL tubes and PCR tubes were respectively heated at 80°C for 30 minutes using the water bath and thermocycler (SimpliAmp™ Thermal Cycler, Applied Biosystems™, ThermoFisher Scientific). After the heating step, 50 μL of each solution was immediately aliquoted from the 5.0 mL tube to each of the duplicate 200 μL tubes. Phosphate buffered saline (PBS) was also included in the PCR tubes as a negative control. All tubes were stored at 4°C overnight. On the following day, tubes were left to equilibrate to room temperature of around 24°C for an hour prior to performing the gelation assay. Biological triplicates were performed in comparing the results of Inversion Assay with the Floating Sphere Assay. The procedure is summarised in Fig 1B.

### Floating sphere assay

Borosilicate glass beads of 3.0 mm (Z143928, Merck KGaA, Germany) were coated with blue ink from Sharpie Permanent Marker Fine Point (Newell Office Brands, USA). The permanent marker was pierced to obtain the ink cartridge. Ink from the cartridge was gently pressed out into a petri-dish. Glass beads were added to the petri dish which was then sealed and shaken vigorously to coat the glass beads evenly with the ink. Density of glass beads was averaged from the mass and radius (Mitutoyo Digimatic Micrometer, Japan) of 10 glass beads assuming spherical dimensions. Using a tweezer, beads were deposited on to the top of the gelling solution. After 5 minutes, the position of the beads was scored as either floating or partially submerged or fully submerged.

## Results and discussion

### A simple miniaturized assay for gelation

Our motivation to develop the Floating Sphere Assay was driven by our observation that inverting 200 μL tubes containing 50 μL of PBS did not result in the solution flowing to the bottom (S1 Fig). For microvolumes such as 50–500 μl, the Inversion Assay is therefore not suitable to detect MGC. Consequently, we developed a gelation assay based on the falling sphere assay which is commonly used to determine viscosities of solutions. By measuring the terminal velocity of a sphere dropped into the solution, the viscosity of the solution can be determined using the Stokes equation. Lower the terminal velocity of the sphere, higher the viscosity of the solution. For our assay, we chose glass beads that have a higher density (2.64 g/cm^3^) than commonly used gelling aqueous solutions (~1 g/cm^3^) to ensure that weight of the sphere overcomes the buoyant forces. We reasoned that if a gel is formed, it might be able to counter the weight of the sphere and thereby prevent it from entering the ‘solution phase’.

To test this above idea, we chose three gelling agents namely gelatin, gellan gum and κ-carrageenan and compared the results obtained with the traditional Inversion Assay. We prepared solutions containing each of the gelling agent at different concentrations in 5 mL tubes and heated the solutions at 80°C in a water bath for 30 min (Fig 1B). To evaluate the possibility of using smaller volumes for gelation, on completion of the heating step, we transferred a small aliquot of the solution from the 5 ml tube into a 0.2 mL tube and then incubated both 5 mL and 200 μL tubes at 4°C overnight to promote gelation. For screens of gelling proteins, it will not be possible to use the water bath for heating since this requires tubes larger than 200 μL but small tube volumes are required to minimise sample volumes. Therefore, to evaluate the potential of heating 200 μL tubes directly, we assessed whether thermocycler is as effective as water bath in heating the gelling agent solutions. In parallel, we prepared 50 μL of the gelling agent solutions in 200 μL tubes and performed the heating step in thermocycler and subsequently stored at 4°C overnight for the gels to set (Fig 1B). After overnight incubation, we equilibrated tubes to room-temperature. Inversion Assay was performed using samples in 5 mL tubes and the Floating Sphere Assay with samples in 200 μL tubes.

Overall, we saw a very good correlation between results from the Inversion and the Floating Sphere Assays for all the three gelling agents. When the bead floated or was partially submerged in the solution in Floating Sphere Assay, gelation was always observed in the Inversion Assay. Solutions in which the bead fully submerged, also did not form gels in the Inversion Assay. The only exception occurred for pH 7 gellan gum where at 0.5%, the bead remained partially submerged in the Floating Sphere Assay but the sample had collapsed in the Inversion Assay. Results from our experiments are presented in Tables 1 – 3.

**Table 1:** Gelation results of gelatin.

	Concentration (%)			
	5.0	2.5	1.25	0.625
(A) Thermocycler–Falling sphere assay	Float	Float and PS	PS	FS
(B) Water bath–Falling sphere assay	Float	Float	PS	FS
(C) Water bath–Inversion assay	Stable	Stable	Stable	Collapsed

**Table 2:** Gelation results of potassium-supplemented κ-carrageenan.

	Concentration (%)						
	2.0	1.5	1.0	0.5	0.25	0.125	0.0625
(A) Thermocycler–Falling sphere assay	Float	Float	Float and PS	Float and PS	PS	PS	FS
(B) Water bath–Falling sphere assay	Float	Float	Float and PS	Float and PS	PS	PS	FS
(C) Water bath–Inversion assay	Stable	Stable	Stable	Stable	Stable	Collapsed	Collapsed

**Table 3:** Gelation results of calcium-supplemented low acyl gellan gum.

	Concentration (%)						
	2.0	1.5	1.0	0.5	0.25	0.125	0.0625
(A) Thermocycler–Falling sphere assay	Float	Float	Float	Float and PS	PS	PS	FS
(B) Water bath–Falling sphere assay	Float	Float	Float and PS	Float and PS	PS	PS	FS
(C) Water bath–Inversion assay	Stable	Stable	Stable	Stable	Stable	Stable	Collapsed

**Fig 2:**
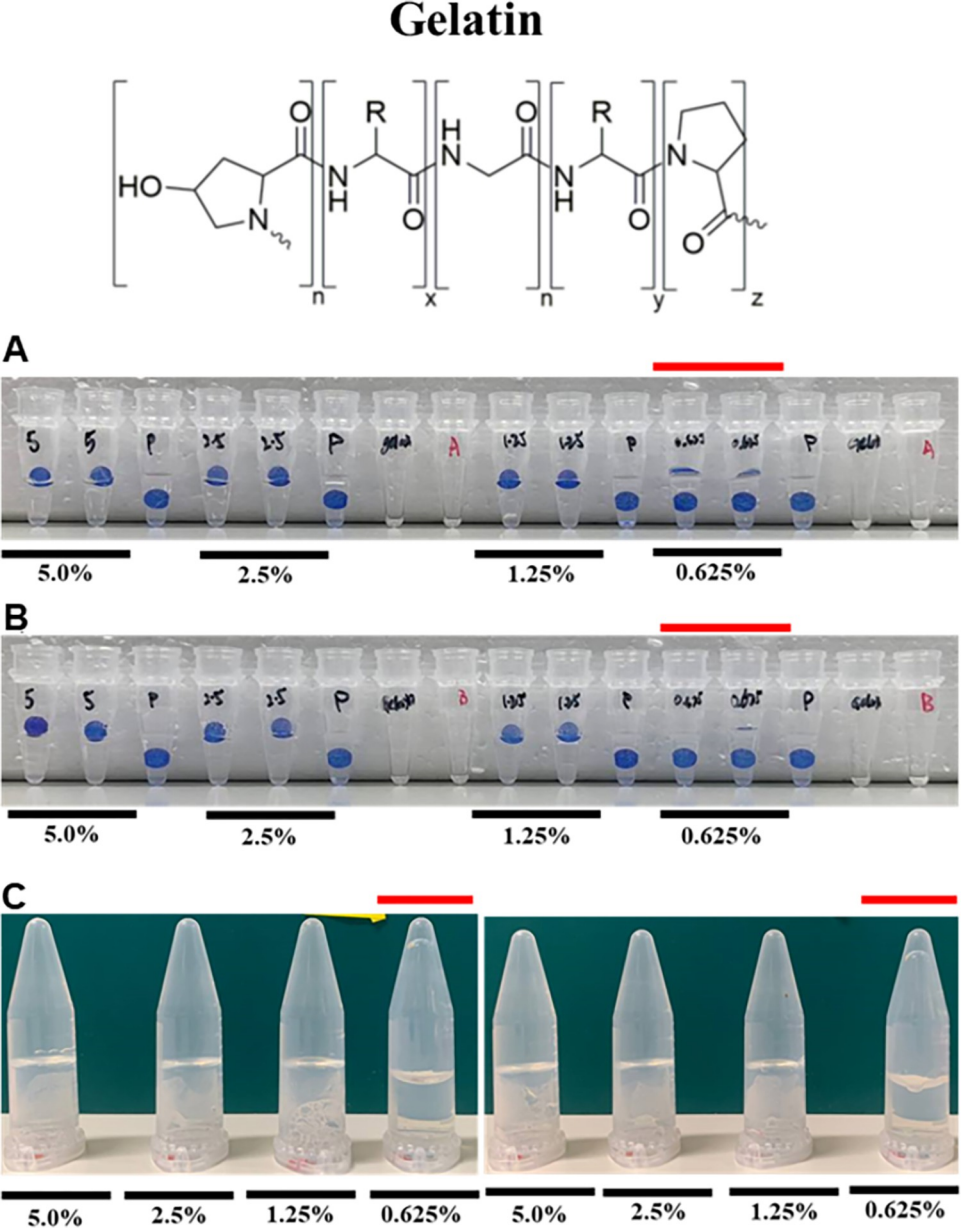
Determining the minimum gelling concentration of gelatin via the floating sphere and inversion assays. Representation of gelation results of gelatin in (a) Thermocycler–Floating Sphere Assay; (b) Water bath–Floating Sphere Assay and (c) Water bath–Inversion Assay. Red line indicates the concentrations below the critical gelling concentration. A minimum of three biological replicates was performed. Results from two additional replicates are presented in S2 Fig.

Gelatin formed a gel at a minimum gelling concentration of 1.25% in both the Floating Sphere and Inversion Assays. Representative images of gelation assays of gelatin (Table 2A) are presented in Fig 2 (Data from two additional biological replicates are presented in S2 Fig). For 1.25–5.0%, the bead either floated or was partially submerged in the Floating Sphere Assay and did not flow in the Inversion Assay. Whereas, in the 0.625% solution of gelatin, the bead was fully submerged in the Floating Sphere Assay and collapsed in the Inversion Assay.

**Fig 3:**
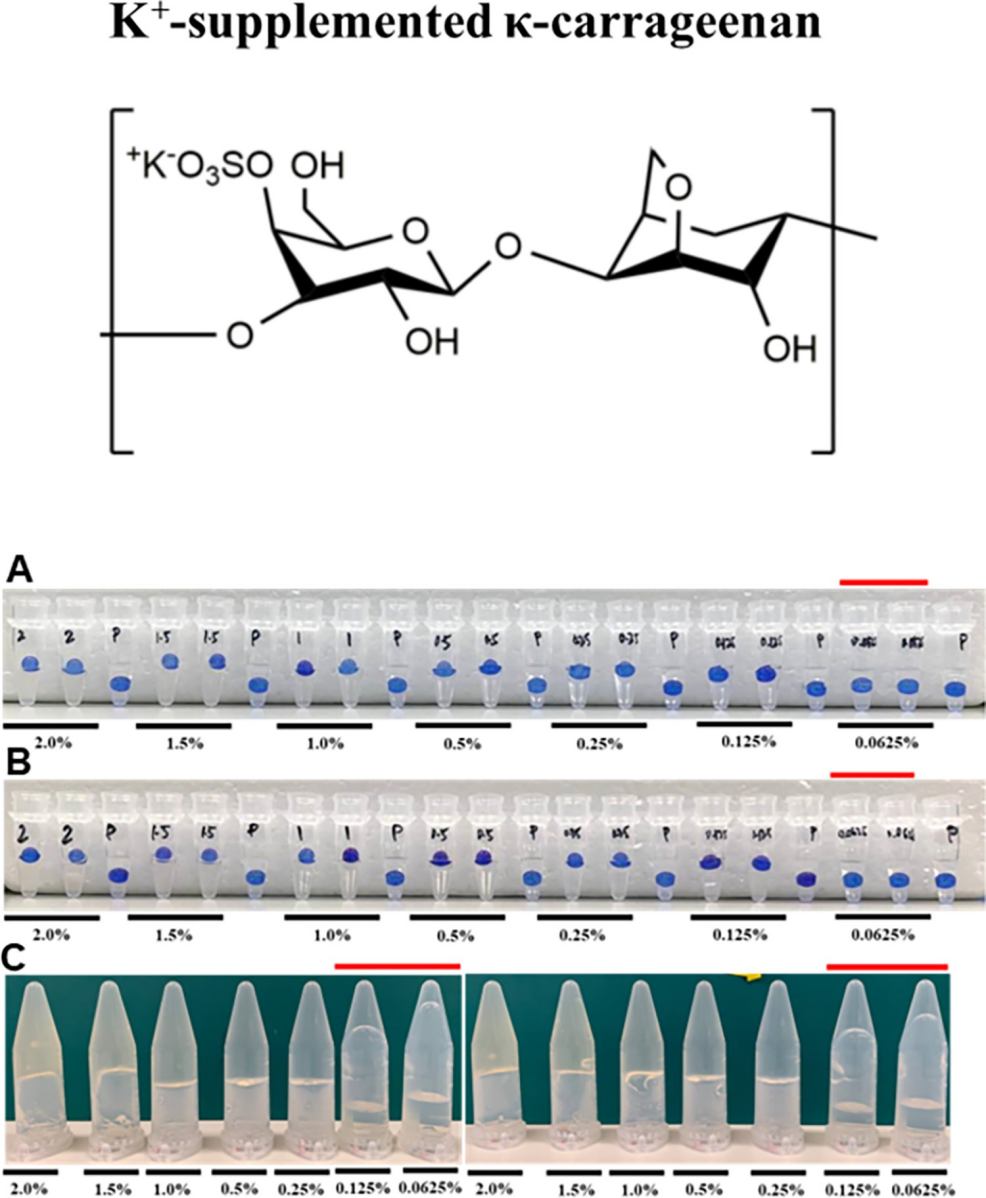
Determining the minimum gelling concentration of potassium-supplemented kappa carrageenan via the floating sphere and inversion assays. Representation of gelation results of potassium-supplemented kappa carrageenan in (a) Thermocycler–Floating Sphere Assay; (b) Water bath–Floating Sphere Assay and (c) water bath–Inversion Assay. Red line indicates the concentrations below the critical gelling concentration. A minimum of three biological replicates was performed. Results from two additional replicates are presented in S3 Fig.

**Fig 4:**
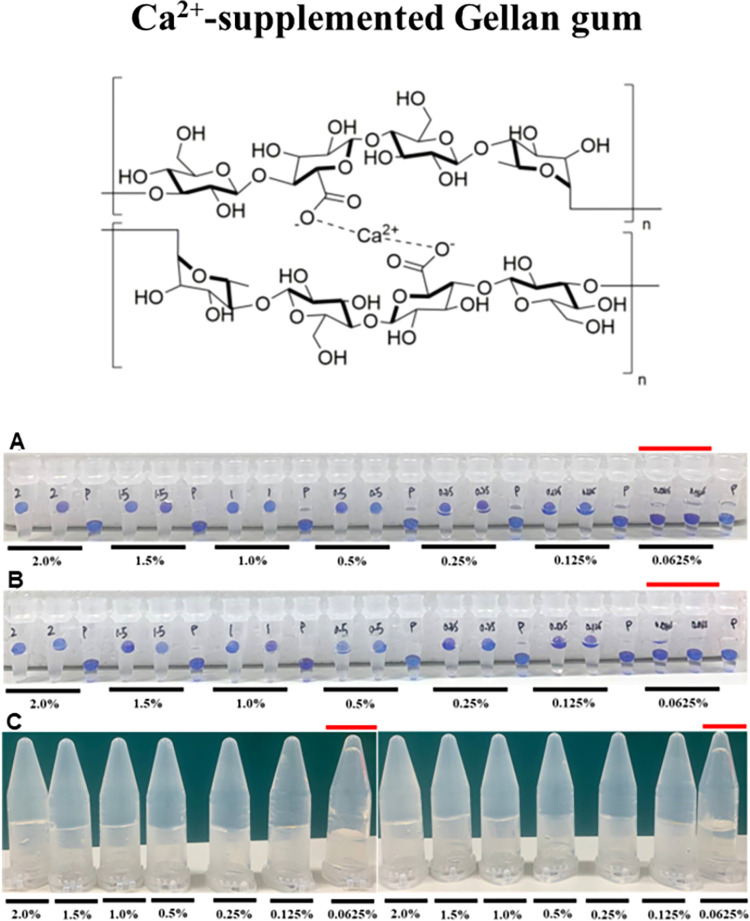
Determining the minimum gelling concentration of calcium-supplemented low acyl gellan gum via the floating sphere and inversion assays. Representation of gelation results of calcium-supplemented low acyl gellan gum in (a) Thermocycler–Floating Sphere Assay; (b) Water bath–Floating Sphere Assay and (c) Water bath–Inversion Assay. Red line indicates the concentrations below the critical gelling concentration. A minimum of three biological replicates was performed. Results from two additional replicates are presented in S4 Fig.

Similar trends were obtained for potassium κ-carrageenan (Table 2) and calcium-supplemented low acyl gellan gum (Table 3) and the data are presented in Figs 3 and 4 respectively (Data from two additional biological replicates are presented in S3 and S4 Figs). For 0.125–2.0% solutions, the bead either floated or was partially submerged in the Floating Sphere Assay and did not flow in the Inversion Assay. Whereas, in the 0.0625% solution, the bead was fully submerged in the falling sphere assay and collapsed in the Inversion Assay (Figs 3 and 4).

Moreover, the values of critical gelling concentrations obtained are largely consistent with values reported in the literature. Critical gelling concentrations was reported to be as low as 0.7% for potassium-supplemented κ-carrageenan solutions [15]; 0.1% [17] and 0.6% for calcium-supplemented low acyl gellan solutions [18]; 0.5% [19] and 1.0% for gelatin [4].

Apart from reduced sample volumes, an additional advantage of the Floating Sphere Assay is its ability to detect the transition zone between strong gels in which the bead floats on top and weak gels in which the bead is partially submerged. This is unlike the conventional Inversion Assay which can only detect the transition zone between gels and non-gelling solutions. This is as reflected from our observations that samples producing weak gels in the Floating Sphere Assay form ‘stable gels’ in the Inversion Assay.

**Fig 5:**
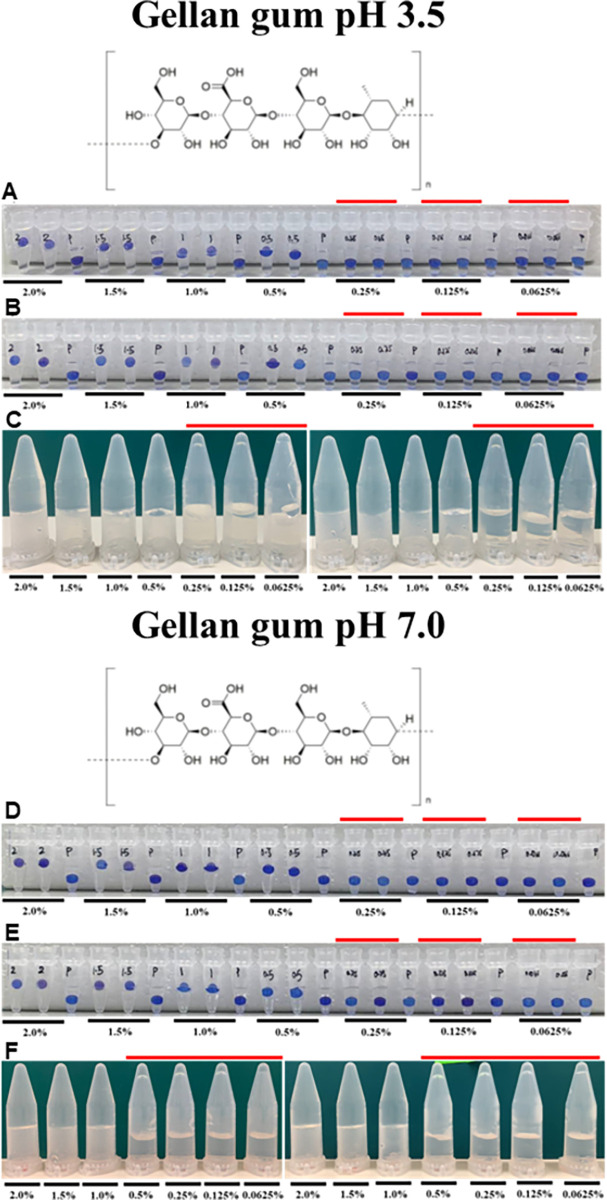
Low acyl gellan gum gels form stronger gels at pH 3.5 compared to pH 7.0. Representation of gelation results of pH 3.5 and pH 7.0 non-supplemented low acyl gellan gum are represented in panels (A)-(C) and (D)-(F respectively. Panels (A) and (D) Thermocycler–Floating Sphere Assay; Panels (B) and (E) Water bath–Floating Sphere Assay; Panels (C) and (F) Water bath–Inversion Assay. Red line indicates the concentrations below the critical gelling concentration. A minimum of 3 biological replicates was performed. Results from two additional replicates are presented in S5 and S6 Figs.

Subsequently, we evaluated the ability of the Floating Sphere Assay to distinguish gels with varying strengths. Several studies have reported that at the same gum concentration in non-calcium supplemented gels, gellan at pH 3.5 would form stronger gels than at pH 7.0. One study reported that rupture stress of the gels at pH 3.5 exceeds that in pH 5.3 and 7.0 counterparts by at least a factor of two [20]. A separate study observed that at pH 3.5, gellan gum set at much lower concentrations than that of pH 5.2 and, 0.5% gels at pH 3.5 produced a gel as strong as a 2.0% counterpart at pH 5.2 [21]. Consistent with these observations, in our Falling Sphere Assay, 1.0% gel corresponds to the transition zone in which beads would be partially submerged in pH 7 gels yet floating at the top in pH 3.5 counterparts (Table 4, Fig 5). Data from two additional biological replicates are presented in S5 and S6 Figs. Therefore, this indicates that our assay has the sensitivity to distinguish gels with varying strengths. This serves as a cost-effective approach to differentiate gel strength as compared to conventional use of mechanical texture analysers and rheometers [20, 21].

**Table 4:** Gelation results of non-supplemented low acyl gellan at pH 3.5 and 7.0.

		Concentration (%)						
		2.0	1.5	1.0	0.5	0.25	0.125	0.0625
(A) Thermocycler and Water bath–Falling sphere assay	pH 3.5	Float	Float	**Float**	PS	FS	FS	FS
	pH 7.0	Float	Float	**PS**	PS	FS	FS	FS
(B)Water bath–Inversion assay	pH 3.5	Stable	Stable	Stable	**Stable**	Collapsed	Collapsed	Collapsed
	pH 7.0	Stable	Stable	Stable	**Collapsed**	Collapsed	Collapsed	Collapsed

### Physical principles underlying the floating sphere assay

**Fig 6:**
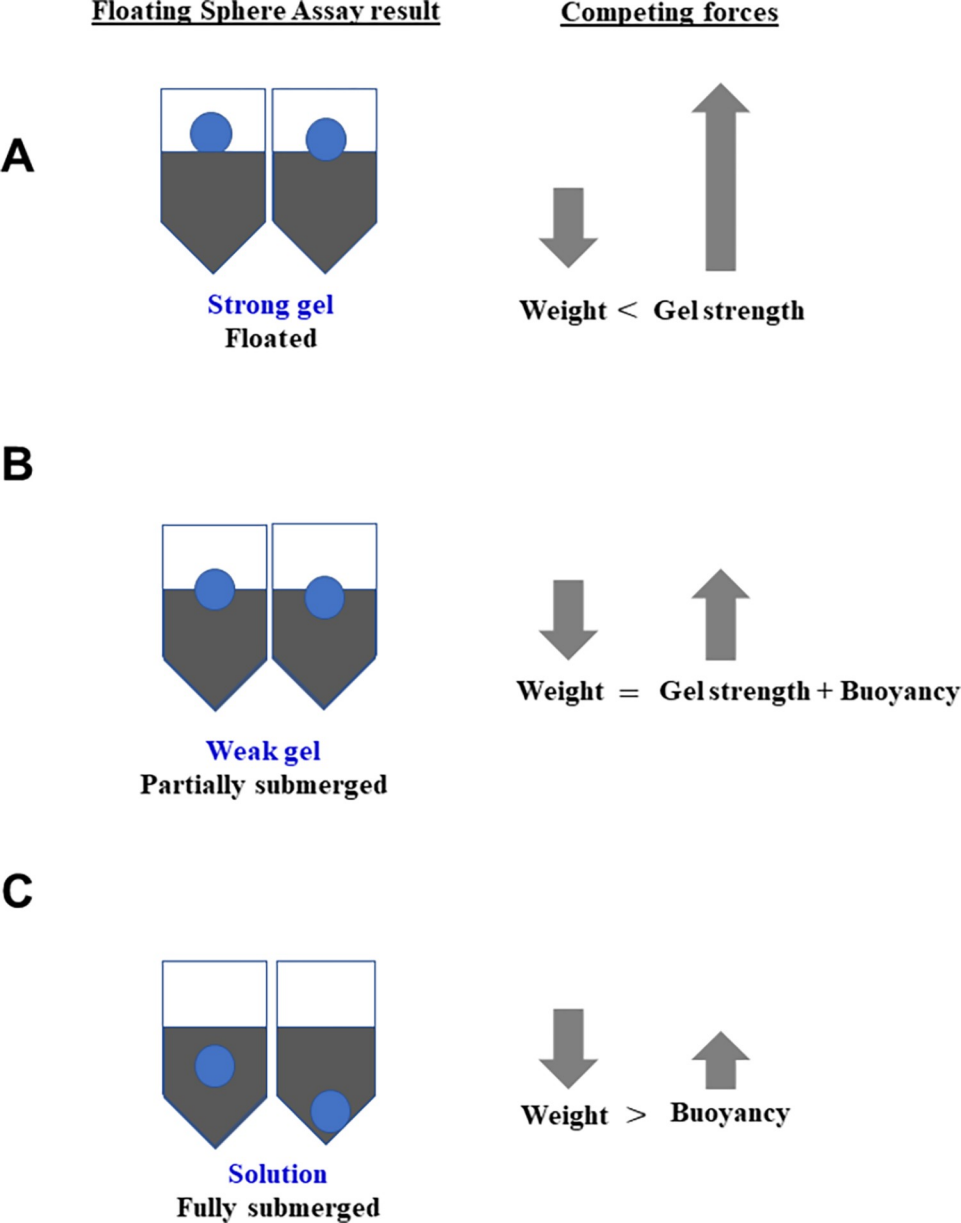
Physical principles underlying the floating sphere assay. Forces acting on the bead for the three possible outcomes of the Floating Sphere assay. (A) Bead is floating; (B) bead is partially submerged; (C) bead is completely submerged. Please see the text for details.

When the glass bead (density = 2.6 g/cm^3^) is dropped into the solution (density = 1 g/cm^3^) in the PCR tube, it behaves in one of the following three ways. 1. **Bead floats**. In this case, the weight of the bead is supported by the strength of the solid gel generated by the intermolecular and intramolecular interactions involving the solvent, gelling agent and additional buffer components (Fig 6A). 2. **Bead is partially submerged.** In this case, the strength of the gel is unable to completely support the weight of the bead. Although the bead exerted a force exceeding rupture stress (if any formed), strength of the gel in combination with the buoyant force due to submerged portion of the bead, matches the weight on the bead, to keep it transiently afloat (Fig 6B). 3. **Bead is fully submerged**. Weight of the bead exceeds the gel strength (if any formed) and enters the solution (Fig 6C).

### Limitations and future work

Our falling sphere assay is technically simple, economical and can be easily adapted for high-throughput screening for gel formation. It might be difficult to distinguish very viscous solutions from weak gels. To differentiate between weak gels and viscous solutions when the bead is partially submerged, lower density beads could be used. This is because the weak gels are more likely to support beads of lower density. Correspondingly, to differentiate strength amongst gels when bead is floating at the top, higher density beads (e.g., steel) might be useful. Floating sphere assay can be used to measure gelation time. Solution of the gelling agent can be placed into multiple tubes. The glass bead can be introduced into tubes at varying times and gelation can be assessed by monitoring the position of the glass bead as described above.

Moreover, this assay could be potentially adapted to facilitate development of a miniaturized texture profile analyser. This would require improved precision of the Floating Sphere Assay, achievable through automating the position at which the ball is deposited into the sample tube. This would serve to standardize the height and angle from which the sphere is dropped, thereby controlling the velocity and area by which the glass bead contacts the surface of the gel solution. This would serve to standardize the depth of depression/compression should the sample be a solid gel and, depth of penetration should the sample be in a liquid-state. Microscopic imaging or reflective-laser based sensing of the former serves can serve as a means to characterize springiness of strong gels (i.e. recovery on compression by a fixed force) while the latter can differentiate viscosity of weaker gels. Lastly, dimensions of the ball used could be further reduced to facilitate the use of even smaller sample volumes to detect gel formation.

## Conclusions

The Floating Sphere Assay is a reliable miniaturised method for gelation with high accuracy to detect MGC and sensitivity to differentiate between strong and weak gels. This can be utilized in high-throughput screens for novel protein gelling agents derived through recombinant or natural extraction approaches.

## Supporting information

S1 Fig Inversion assay does not work with microvolumes of test solution. Inverted PCR tubes containing PBS solution. Solution level is marked by black arrows.
(TIF)

S2 Fig Determining the minimum gelling concentration of gelatin via the floating sphere and inversion assays. Data from two additional replicates for gelation results of gelatin are presented in (A)–(C) and (D)–(F) respectively. (A) and (D) correspond to Thermocycler–Floating Sphere Assay; (B) and (E) correspond to Water bath–Floating Sphere Assay; (C) and (F) correspond to Water bath–Inversion Assay.
(TIF)

S3 Fig Determining the minimum gelling concentration of potassium-supplemented kappa carrageenan via the floating sphere and inversion assays. Data from two additional replicates for gelation results of potassium-supplemented kappa carrageenan are presented in (A)–(C) and (D)–(F) respectively. (A) and (D) correspond to Thermocycler–Floating Sphere Assay; (B) and (E) correspond to Water bath–Floating Sphere Assay; (C) and (F) correspond to Water bath–Inversion Assay.
(TIF)

S4 Fig Determining the minimum gelling concentration of calcium-supplemented low acyl gellan gum via the floating sphere and inversion assays. Data from two additional replicates for gelation results of calcium-supplemented low acyl gellan gum are presented in (A)–(C) and (D)–(F) respectively. (A) and (D) correspond to Thermocycler–Floating Sphere Assay; (B) and (E) correspond to Water bath–Floating Sphere Assay; (C) and (F) correspond to Water bath–Inversion Assay.
(TIF)

S5 Fig Low acyl gellan gum gels form stronger gels at pH 3.5 compared to pH 7.0. Data from two additional replicates for gelation results of low acyl gellan gum gels at pH3.5 are presented in (A)–(C) and (D)–(F) respectively. (A) and (D) correspond to Thermocycler–Floating Sphere Assay; (B) and (E) correspond to Water bath–Floating Sphere Assay; (C) and (F) correspond to Water bath–Inversion Assay.
(TIF)

S6 Fig Low acyl gellan gum gels form stronger gels at pH 3.5 compared to pH 7.0. Data from two additional replicates for gelation results of low acyl gellan gum gels at pH 7.0 are presented in (A)–(C) and (D)–(F) respectively. (A) and (D) correspond to Thermocycler–Floating Sphere Assay; (B) and (E) correspond to Water bath–Floating Sphere Assay; (C) and (F) correspond to Water bath–Inversion Assay.
(TIF)

## References

[1] Makki K, Deehan EC, Walter J, Bäckhed F. The Impact of Dietary Fiber on Gut Microbiota in Host Health and Disease. Cell Host & Microbe. 2018;23(6):705–15. doi: 10.1016/j.chom.2018.05.012 29902436

[2] MacQueen LA, Alver CG, Chantre CO, Ahn S, Cera L, Gonzalez GM, et al. Muscle tissue engineering in fibrous gelatin: implications for meat analogs. npj Science of Food. 2019;3(1):20–. doi: 10.1038/s41538-019-0054-8 31646181 PMC6803664

[3] Alipal J, Mohd Pu’ad NAS, Lee TC, Nayan NHM, Sahari N, Basri H, et al. A review of gelatin: Properties, sources, process, applications, and commercialisation. Materials Today: Proceedings. 2021;42:240–50. 10.1016/j.matpr.2020.12.922.

[4] Djabourov M, Lechaire J-P, Gaill F. Structure and rheology of gelatin and collagen gels. Biorheology. 1993;30:191–205. doi: 10.3233/bir-1993-303-405 8286722

[5] Aydemir LY, Yemenicioğlu A. Potential of Turkish Kabuli type chickpea and green and red lentil cultivars as source of soy and animal origin functional protein alternatives. Lebensmittel-Wissenschaft + [ie und] Technologie: Elsevier Ltd; 2013.

[6] Yang X, Nisar T, Liang D, Hou Y, Sun L, Guo Y. Low methoxyl pectin gelation under alkaline conditions and its rheological properties: Using NaOH as a pH regulator. Food Hydrocolloids. 2018;79:560–71. 10.1016/j.foodhyd.2017.12.006.

[7] Hughes MP, Sawaya MR, Boyer DR, Goldschmidt L, Rodriguez JA, Cascio D, et al. Atomic structures of low-complexity protein segments reveal kinked β sheets that assemble networks. Science. 2018;359(6376):698–701. doi: 10.1126/science.aan6398 29439243 PMC6192703

[8] Ali SH, Al-Zuky AAD, Al-Saleh AH, Mohamad HJ. Measure liquid viscosity by tracking falling ball Automatically depending on image processing algorithm. Journal of Physics: Conference Series. 2019;1294(2):22002–. doi: 10.1088/1742-6596/1294/2/022002

[9] Tang JX. Measurements of fluid viscosity using a miniature ball drop device. Review of Scientific Instruments. 2016;87(5):54301–. doi: 10.1063/1.4948314 27250443

[10] Rockwell MA, Fechheimer M, Taylor DL. A comparison of methods used to characterize gelation of actin in vitro. Cell Motility. 1984;4(3):197–213. doi: 10.1002/cm.970040305 6744386

[11] Sgreva NR, Davaille A, Kumagai I, Kurita K. Interaction between a falling sphere and the structure of a non-Newtonian yield-stress fluid. Journal of Non-Newtonian Fluid Mechanics. 2020;284:104355–. 10.1016/j.jnnfm.2020.104355.

[12] Wang J, Reitz F, Donaldson T, Pagliaro L. A computer-interfaced falling ball viscometer. Journal of Biochemical and Biophysical Methods. 1994;28(4):251–61. doi: 10.1016/0165-022x(94)90001-9 7963247

[13] Pang Z, Deeth H, Sopade P, Sharma R, Bansal N. Rheology, texture and microstructure of gelatin gels with and without milk proteins. Food Hydrocolloids. 2014;35:484–93. 10.1016/j.foodhyd.2013.07.007.

[14] Morris ER, Nishinari K, Rinaudo M. Gelation of gellan–A review. Food Hydrocolloids. 2012;28(2):373–411. 10.1016/j.foodhyd.2012.01.004.

[15] Chen Y, Liao M-L, Dunstan DE. The rheology of K+-κ-carrageenan as a weak gel. Carbohydrate Polymers. 2002;50(2):109–16. 10.1016/S0144-8617(02)00009-7.

[16] Hermansson AM, Eriksson E, Jordansson E. Effects of potassium, sodium and calcium on the microstructure and rheological behaviour of kappa-carrageenan gels. Carbohydrate Polymers. 1991;16(3):297–320. 10.1016/0144-8617(91)90115-S.

[17] Lochhead RY. Chapter 13—The Use of Polymers in Cosmetic Products. In: Sakamoto K, Lochhead RY, Maibach HI, Yamashita Y, editors. Cosmetic Science and Technology. Amsterdam: Elsevier; 2017. p. 171–221.

[18] Tang J, Lelievre J, Tung MA, Zeng Y. Polymer and Ion Concentration Effects on Gellan Gel Strength and Strain. Journal of Food Science. 1994;59(1):216–20. 10.1111/j.1365-2621.1994.tb06934.x.

[19] Pandey VN, Tiwari N, Pandey VS, Rao A, Das I. Chapter 13—Targeted drug delivery and gene therapy through natural biodegradable nanostructures in pharmaceuticals. In: Grumezescu AM, editor. Nanoarchitectonics in Biomedicine: William Andrew Publishing; 2019. p. 437–72.

[20] Picone CSF, Cunha RL. Influence of pH on formation and properties of gellan gels. Carbohydrate Polymers. 2011;84(1):662–8. 10.1016/j.carbpol.2010.12.045.

[21] Cassanelli M, Prosapio V, Norton I, Mills T. Acidified/basified gellan gum gels: The role of the structure in drying/rehydration mechanisms. Food Hydrocolloids. 2018;82:346–54. 10.1016/j.foodhyd.2018.04.024.

