# Supplementary figures and images for: Floating sphere assay: A rapid qualitative method for microvolume analysis of gelation
Source: PLoS One. 2022 Jul 8;17(7):e0266309. doi: 10.1371/journal.pone.0266309 (PMC9269766; doi:10.1371/journal.pone.0266309)

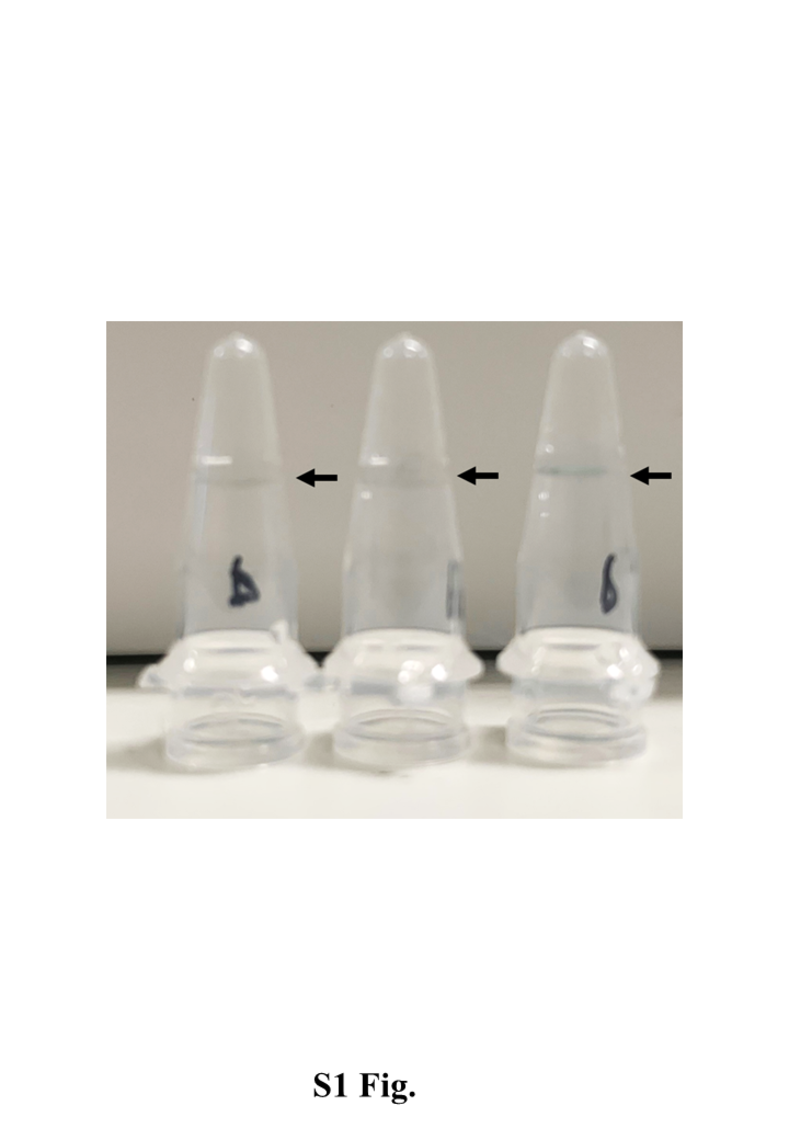

Supplement: S1 Fig — Inverted PCR tubes containing PBS solution. Solution level is marked by black arrows. (TIF) [file pone.0266309.s001.tif]

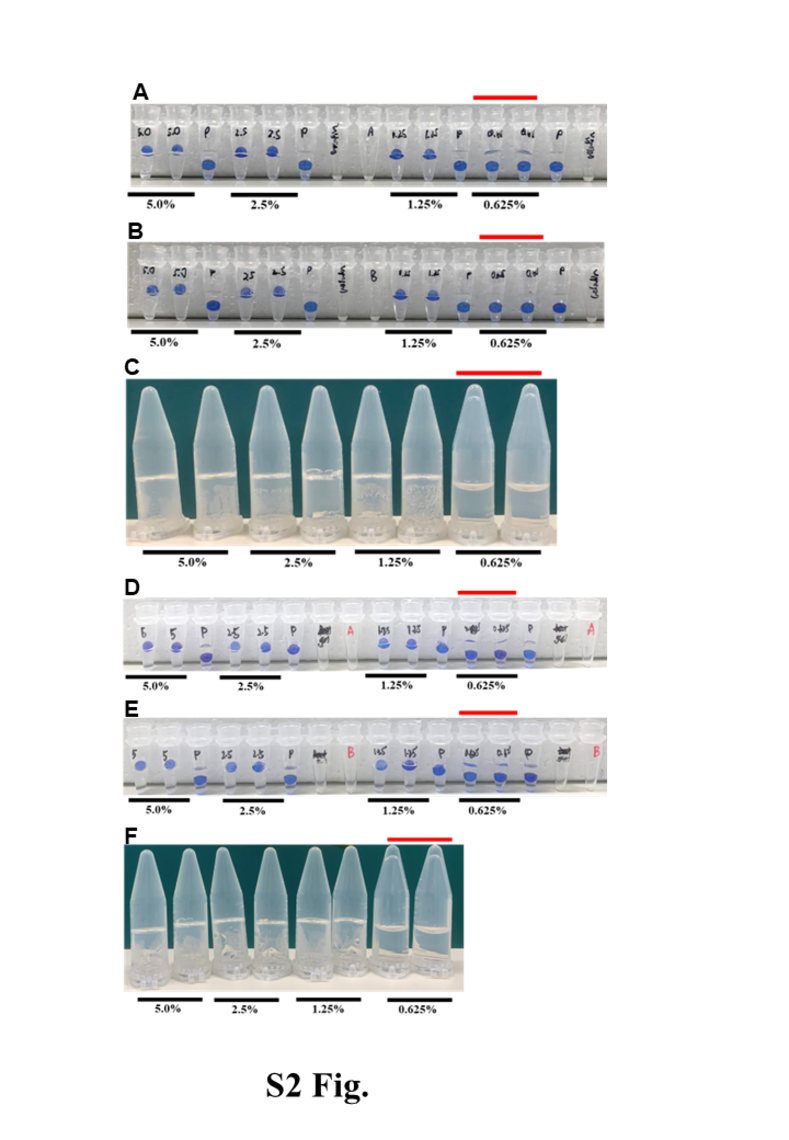

Supplement: S2 Fig — Data from two additional replicates for gelation results of gelatin are presented in (A)–(C) and (D)–(F) respectively. (A) and (D) correspond to Thermocycler–Floating Sphere Assay; (B) and (E) correspond to Water bath–Floating Sphere Assay; (C) and (F) correspond to Water bath–Inversion Assay. (TIF) [file pone.0266309.s002.tif]

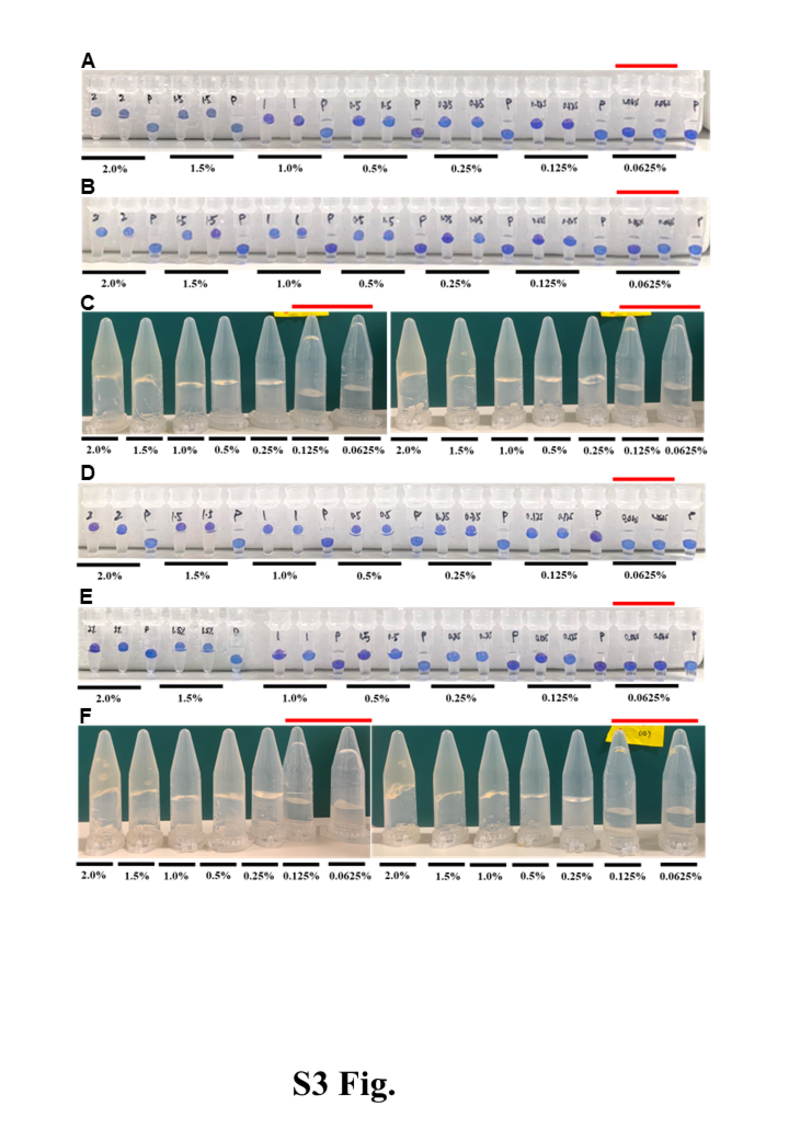

Supplement: S3 Fig — Data from two additional replicates for gelation results of potassium-supplemented kappa carrageenan are presented in (A)–(C) and (D)–(F) respectively. (A) and (D) correspond to Thermocycler–Floating Sphere Assay; (B) and (E) correspond to Water bath–Floating Sphere Assay; (C) and (F) correspond to Water bath–Inversion Assay. (TIF) [file pone.0266309.s003.tif]

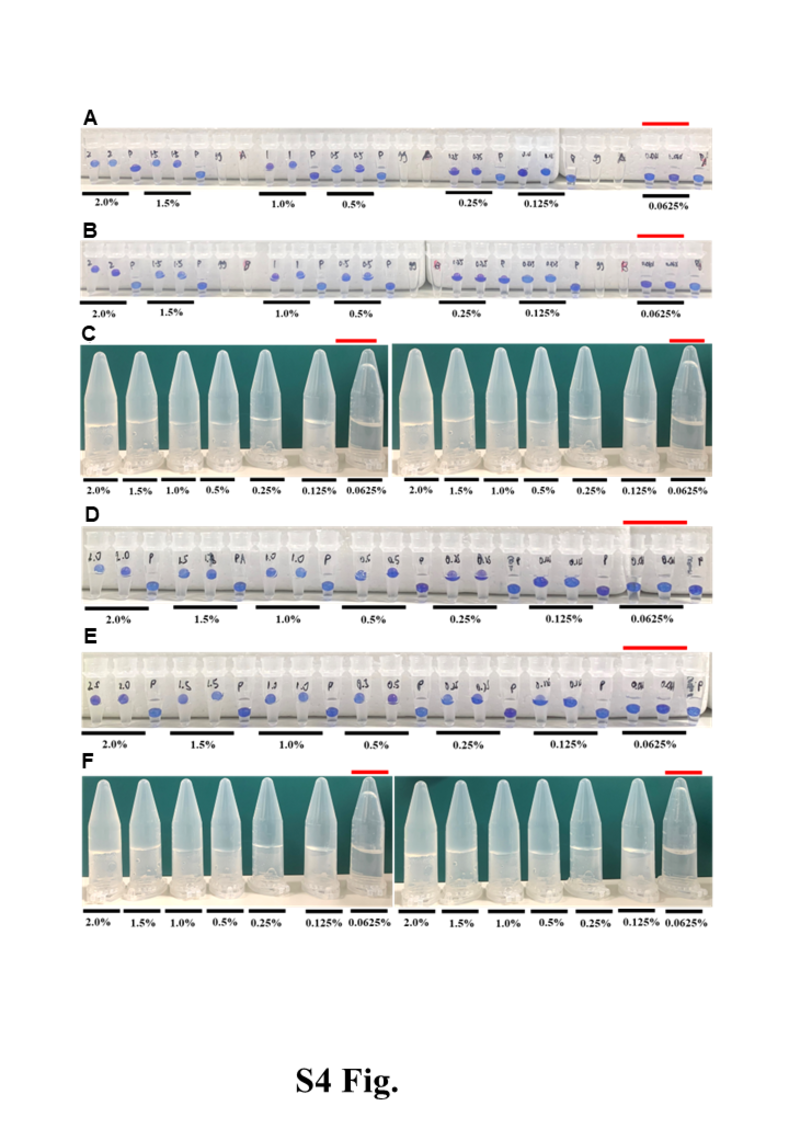

Supplement: S4 Fig — Data from two additional replicates for gelation results of calcium-supplemented low acyl gellan gum are presented in (A)–(C) and (D)–(F) respectively. (A) and (D) correspond to Thermocycler–Floating Sphere Assay; (B) and (E) correspond to Water bath–Floating Sphere Assay; (C) and (F) correspond to Water bath–Inversion Assay. (TIF) [file pone.0266309.s004.tif]

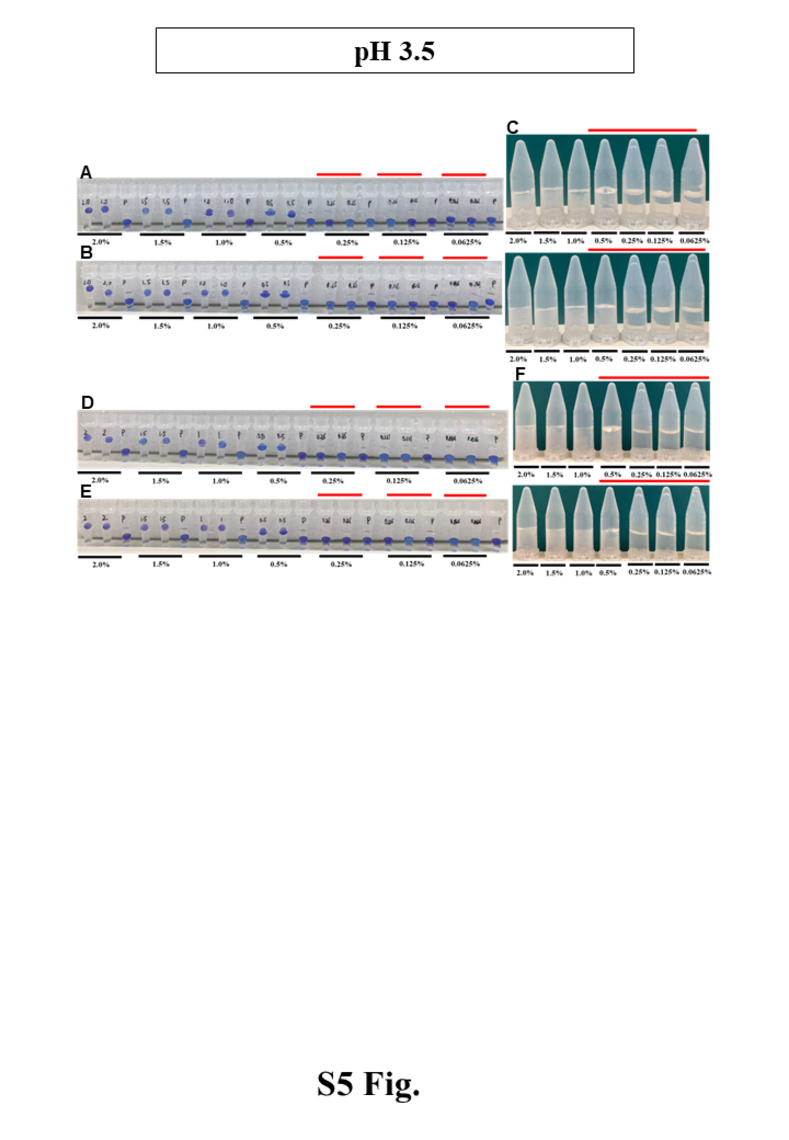

Supplement: S5 Fig — Data from two additional replicates for gelation results of low acyl gellan gum gels at pH3.5 are presented in (A)–(C) and (D)–(F) respectively. (A) and (D) correspond to Thermocycler–Floating Sphere Assay; (B) and (E) correspond to Water bath–Floating Sphere Assay; (C) and (F) correspond to Water bath–Inversion Assay. (TIF) [file pone.0266309.s005.tif]

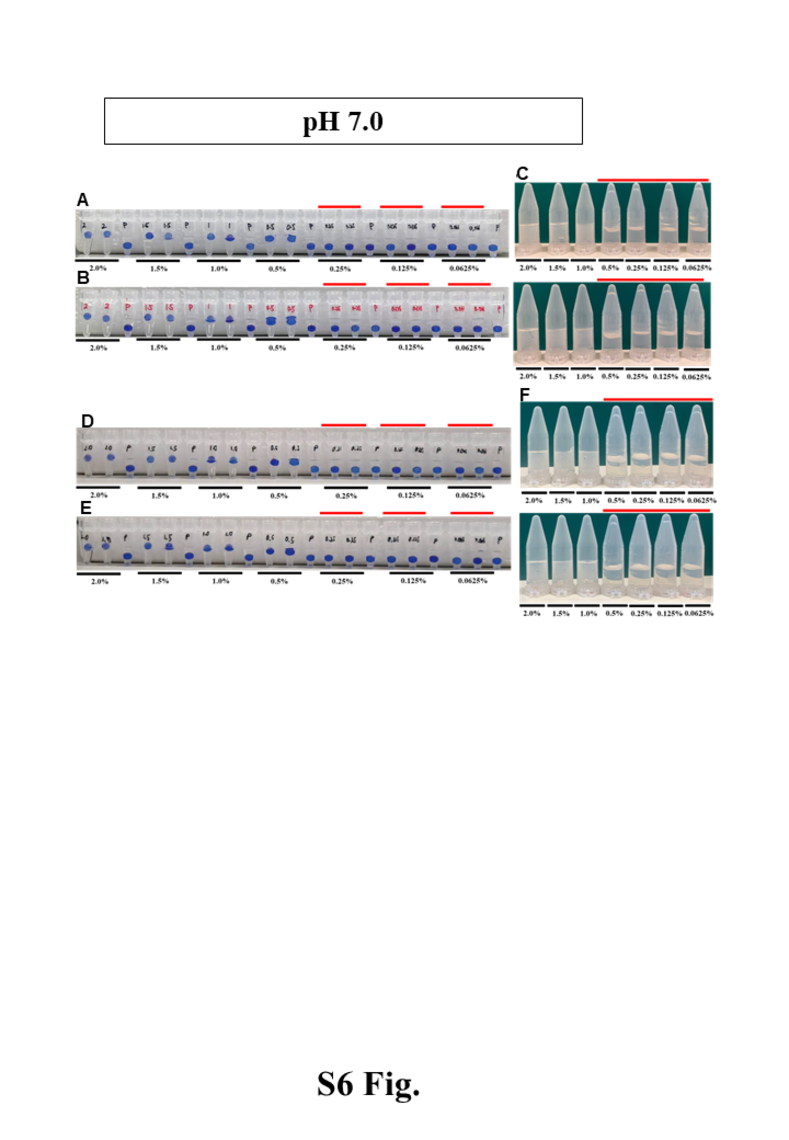

Supplement: S6 Fig — Data from two additional replicates for gelation results of low acyl gellan gum gels at pH 7.0 are presented in (A)–(C) and (D)–(F) respectively. (A) and (D) correspond to Thermocycler–Floating Sphere Assay; (B) and (E) correspond to Water bath–Floating Sphere Assay; (C) and (F) correspond to Water bath–Inversion Assay. (TIF) [file pone.0266309.s006.tif]
